# Psychometric Properties of the Insomnia Severity Index Among Arabic Chronic Diseases Patients

**DOI:** 10.1177/23779608221107278

**Published:** 2022-06-23

**Authors:** Mohammed Al Maqbali, Norah Madkhali, Geoffrey L. Dickens

**Affiliations:** 1 Department of Nursing Midwifery and Health, 5995 Northumbria University, Newcastle-Upon-Tyne, UK; 2 123285 Jazan University, Saudi Arabia; 3 Mental Health Nursing Department of Nursing, 373117 Midwifery and Health Faculty of Health and Life Sciences, Northumbria University, Newcastle-Upon-Tyne, UK; 4 Western Sydney University, Sydney, Australia

**Keywords:** ISI, validity, reliability, Arabic, PCA, CFA

## Abstract

**Introduction:**

The Insomnia Severity Index (ISI) is a self-administrated questionnaire most frequently used to assess insomnia in clinical and non-clinical populations.

**Objective:**

To evaluate the psychometric properties of the Arabic ISI among patients diagnosed with chronic diseases.

**Methods:**

A cross-sectional and descriptive correlational design was used. A total of 1,005 patients with chronic diseases completed the seven items of the Arabic ISI version. The scale was assessed in terms of acceptability, internal consistency, and validity. Construct validity was explored with the use of principal factor analysis and confirmatory factor analysis, to examine the dimensional structure of the ISI.

**Results:**

The Cronbach's alpha coefficient for the Arabic ISI was 0.82, which shows good reliability. The total ISI score did not have floor or ceiling effects. There was evidence of discriminate validity. The Principal Component Analysis (PCA) indicated two factors (four items loading on Factor I and three items loading on Factor II). The construct validity of PCA in terms of two factors was explored by confirmatory factor analysis to examine the dimensional structure of the ISI. The confirmatory factor analysis showed an absolute fit for the two-factor model.

**Conclusion:**

The results support the two-factor structure of ISI. The Arabic version of the ISI demonstrated good reliability and validity for assessing insomnia in patients diagnosed with chronic diseases.

## Introduction

Patients diagnosed with chronic conditions such as diabetes and heart disease often experience insomnia, a feature that can continue until the end of the individual's life (Bean et al., 2021; Frøjd et al., 2021; Li et al., 2021). It is estimated that insomnia affects 36% of heart disease patients (Da Costa et al., 2017), and 39% of diabetic patients (Koopman et al., 2020). Insomnia in patients with a chronic condition has a significant negative impact on their quality of life (Morlock & Dobrescu, 2018), which might increase the risk of morbidity and mortality (Lovato & Lack, 2019). In addition, insomnia has been associated with cancer recurrence (Robertson et al., 2016), and may result in delays with regard to wound healing (Smith et al., 2018), increased cognitive dysfunction (Olaithe et al., 2021), and reduced work productivity (Kayaba et al., 2021).

One of the most widely-used instruments for measuring insomnia in both clinical and non-clinical populations is the Insomnia Severity Index (ISI). The scale is easy to understand, can be completed within 3 to 5 min, and contains seven items. The original English ISI was used to assess sleep quality in clinical patients with sleep disorders. The instrument exhibits good validity and test-retest reliability (Bastien et al., 2001).

The ISI has been used across a variety of clinical populations such as those with rheumatoid arthritis (Sellami et al., 2021), diabetes (Alshehri et al., 2020), cardiovascular conditions (Siebmanns et al., 2021), and cancer (Schulte et al., 2021). The ISI has been translated into different languages, including Hindi (Lahan & Gupta, 2011), Swedish (Angelhoff et al., 2020), Chinese (Shapour & Gang, 2013), and Korean (Cho et al., 2014), all versions exhibiting the good psychometric properties. In addition, the ISI has been translated and validated with an Arabic sample living in the United States (Suleiman & Yates, 2011). However, whilst it is acknowledged that Arabic countries share a common language with this US sample, there are cultural differences that need to be taken into consideration. In addition, the validation study of the US-Arabic version of the ISI used a non-clinical sample (Suleiman & Yates, 2011).

To the best of our knowledge, the Arabic ISI has not been validated with clinical samples in Arabic countries. Therefore, the aim of this study is to examine the validity and reliability of the Arabic ISI with a heterogeneous sample of chronic illness patients. The validation of the Arabic ISI in an Arabic country will provide evidence for it as a suitable and acceptable cultural fit instrument for the screening and assessment of insomnia in clinical practice.

## Methods

### Participants

A cross-sectional survey design was used, and participants were recruited from the three-outpatient clinics in Jazan, Saudi Arabia. Data was collected from August 2021 to October 2021. Inclusion criteria for participation were as follows: adult patients over 18 years of age, able to speak and write in Arabic, and no known psychiatric or neurological disorders that could interfere with study participation. They must be diagnosed with one or more of chronic diseases. An exclusion criterion for the participants was being a patient below 18 years of age.

### Instrument

The ISI is a self-completed questionnaire used to assess insomnia occurring over the previous two weeks (Bastien et al., 2001). The scale has seven items; the first three items assess the severity of difficulties with falling sleep (item 1a), maintaining sleep (item 1b), and early morning awakening (item 1c). The last four items capture satisfaction with the current sleep pattern (item 2), interference with daily functioning (item 3), noticeability of impairment (item 4), and degree of distress caused by the sleep problem (item 5). The scores of each of the seven items range from 0 to 4 (0  =  none; 4 = very severe), and a total score can be calculated by summing the seven items, giving a range from 0 to 28, with higher scores indicating greater insomnia severity. Total scores are interpreted as 0–7, absence of insomnia; 8–14, sub-threshold insomnia; 15–21, moderate insomnia; 22–28, severe insomnia (Bastien et al., 2001).

Permission was given by the authors to use the ISI instrument (Suleiman & Yates, 2011). Demographic and clinical data was collected on age, gender, educational level, marital status, employment status, comorbidities, and time since diagnosis.

### Statistical Analyses

Data were analyzed using the Statistical Package for the Social Sciences (SPSS) 25.0. The internal consistency of the ISI was evaluated using Cronbach's alpha coefficient for each subscale, and the overall scale, with an alpha of 0.70 or higher, was considered to be acceptable (Nunnally, 1994). In addition, the Cronbach's alpha of items’ total correlations and the total ISI score will be compared with (Bastien et al., 2001) the original validation of the ISI.

Floor and ceiling effects were evaluated by examining the number of individuals in the total sample who achieved the lowest or highest scores on the scales; these were deemed of importance if more than 15% of the respondents achieved the lowest or highest possible scores (Lim et al., 2015). The discriminant validity of the ISI was tested using one-way analysis of variance (ANOVA), and independent sample t-tests were used to test the differences in the ISI mean scores between gender, cancer site, and comorbidities.

First, the sample was divided using an algorithm in SPSS V 25 which calculates two random samples, each made up of approximately 50% of the total number of respondents. The first sample derived in this way (*n*  =  504) was for principal component analysis (PCA) while the second was used as a replication sample (*n*  =  501) for confirmatory factor analysis (CFA).

Factor structure was tested by using PCA with a Varimax rotation. This was conducted using seven items for initial estimation. Eigenvalues (≥ 1) and a scree plot test guided factor retention. The stability of the data was tested using the Kaiser-Meyer-Olkin Test (KMO ˃ 0.5) (Kaiser, 1970) and Bartlett's statistic (Bartlett, 1950). A factor loading of greater than 0.40 was returned (Horn, 1965; Kline, 2015). PCA was conducted prior to CFA in order to investigate the factor structure in this sample.

CFA was conducted using AMOS 25 to examine the fit statistics of the model created by the PCA by using the replication sample (*n*  =  501). In addition, the ISI was originally specified as a three-factor model (Factor I “Impact”: items 3, 4, and 5; Factor II “Severity”: items 1a, 1b, and 1c; Factor III “Satisfaction”: items 1a, 2, and 5) in English (Bastien et al., 2001). Therefore, CFA was conducted using AMOS 25 to examine the fit statistics of the two models.

The following criteria were used to evaluate the model fit: non-significance of chi-square (χ^2^-test); chi-square per degree of freedom (χ^2^/*df*) of less than 3; Root Mean Square Error of Approximation (RMSEA) of less than or equal to 0.08; Comparative Fit Index (CFI) greater than 0.95; the Goodness of Fit Index (GFI), and Adjusted Goodness of Fit Index (AGFI) of more than or equal to 0.90 to indicate good fit; and the Tucker-Lewis Index (TLI) greater than 0.90 (Hair et al., 2014).

## Results

The demographic and clinical characteristics of the participants are presented in Table 1. In terms of gender, 52.5% were female, and 47.5% were male. The largest age group was 18–30 years (29%). Approximately 60% had more than one chronic disease, 24.5% were diabetic, and 15.1% had a heart disease or were hypertensive. The ISI total scores ranged from 0 to 28 with a mean of 11.39 (SD 5.75). Using the cut-off criteria of the ISI total score as suggested by Bastien et al., 2001, 536 (53%) had sub-threshold insomnia, 195 (19.4%) had moderate insomnia, and 50 (5%) had severe insomnia.

**Table 1.** Descriptive Statistics of the Demographic and Clinical Characteristics of the Participant (*N*  =  1,005).

Variables	*n*	%
**Gender**		
Male	477	47.5
Female	528	52.5
**Age**		
18–30	291	29
31–40	113	11.2
41–50	188	18.7
51–60	191	19
>60	222	22.1
**Marital status**		
Married	649	64.6
Single	286	28.5
Divorced/separated/widowed	70	7
**Education level**		
Basic education	348	34.6
Secondary education	338	33.6
Degree	319	31.7
**Employment status**		
Employed	249	24.8
Retired	208	20.7
Unemployed	545	54.2
**Comorbidities**		
Diabetic	250	24.9
Heart disease/hypertensive	152	15.1
Two or more comorbidities	603	60
**Month since diagnosis**		
≤6 months	199	19.8
>6 months	806	80.2

### Reliability

The ISI total score did not exhibit floor or ceiling effects for the Arabic chronic diseases’ patients. However, five items did demonstrate floor effects. The floor effect was observed with regard to terminal, satisfaction, interference, noticeability, and distress, which indicated that more than 15% of participants achieved the lowest score.

Internal consistency was assessed using Cronbach's alpha, with the ISI Arabic version score being 0.82 which was considered to be acceptable. All seven items appeared to measure a particular aspect of the ISI. The largest component-total correlation coefficients were found for the distress item (0.65), whereas the smallest was found for the terminal item (0.50). The Cronbach's alpha of items’ total correlations and the total ISI score was compared with the original validation of the ISI (Table 2).

**Table 2.** Internal Consistency. Cronbach's α and Ceiling-Floor Effect for ISI Items and Total Score (*N*  =  1,005).

		Scores		Ceiling effect (%)	Floor effect (%)	Corrected item-total correlation	Cronbach's Alpha if item deleted	Cronbach's
Items	Score Range	Mean	SD					
1a: Initial	0-4	1.88	1.15	7.9	13.3	0.52	0.80	0.36*
1b: Middle	0-4	1.80	1.14	6.5	12.5	0.52	0.80	0.57*
1c: Terminal	0-4	1.75	1.17	6.0	16.2	0.50	0.81	0.52*
2: Satisfaction	0-4	1.64	1.16	7.0	20.3	0.58	0.79	0.42*
3: Interference	0-4	1.52	1.26	8.7	25.2	0.56	0.79	0.67*
4: Noticeability	0-4	1.49	1.23	8.1	25.8	0.59	0.79	0.59*
5: Distress	0-4	1.30	1.19	5.8	30.8	0.65	0.78	0.52*
Total score	0–28	11.39	5.75	1.7	4.5	.82		0.74*

*Bastien et al., 2001.

### Discriminant Validity

The differences between the mean of global PSQI in terms of gender, age, employment status, and comorbidities, are shown in Table 3. Of the four discriminative hypotheses, three were accepted (*p *< .05). This indicated that the Arabic ISI scale has discrimination validity.

**Table 3.** Discriminative Validity of the Insomnia Severity Index Arabic Version.

	*n*	Mean	SD	*F*	*p*
**Gender**				3.33	.005
Male	477	10.86	5.46		
Female	528	11.86	5.96		
**Age**				1.35	.25
18–30	291	11.63	5.44		
31–40	113	10.73	5.55		
41–50	188	11.43	5.70		
51–60	191	10.81	5.78		
> 60	222	11.86	6.23		
**Employment status**				8.65	.00
Employed	249	10.61	5.32		
Retired	208	10.51	5.17		
Unemployed	545	12.07	6.07		
**Comorbidities**				19.55	.00
Diabetic	250	9.63	6.19		
Heart disease/hypertensive	152	13.04	4.97		
More than one comorbidity	603	11.7	5.57		

### Factorial Validity

Component analyses. A PCA with orthogonal rotation (varimax method) was completed on seven ISI items for the first derivation sample (*n*  =  504). The KMO of sampling adequacy was 0.80, and Bartlett's Test of Sphericity (*p* < .001) with regard to the sample met the condition of factor analyses. The cumulative variances rule were above 40. A PCA with orthogonal rotation (varimax method) revealed two factors with eigenvalues greater than 1 (3.26 for Factor I; 1.5 for Factor II).

The scree plot supported a two-factor solution, accounting for 67.98% of the total variance (Factor I  =  46.53%; Factor II  =  21.45%). The first factor corresponded to satisfaction with current sleep pattern (item 2), interference with daily functioning (item 3), noticeability of impairment (item 4), and degree of distress caused by the sleep problem (item 5), while the second factor reflected falling asleep (item 1a), maintaining sleep (item 1b) and early morning awakening (item 1c) (see Table 4).

**Table 4.** Factor Loadings Principal Component Analysis With Varimax Rotation for ISI Items (*n*  =  501).

	Factor I	Factor II	Communality
1a: Initial		0.776	0.647
1b: Middle		0.866	0.765
1c: Terminal		0.815	0.679
2: Satisfaction	0.766		0.620
3: Interference	0.802		0.660
4: Noticeability	0.819		0.678
5: Distress	0.820		0.710
Variance %	46.530	21.45	67.98
Cronbach's alpha	.83	.78	

**Figure 1.**
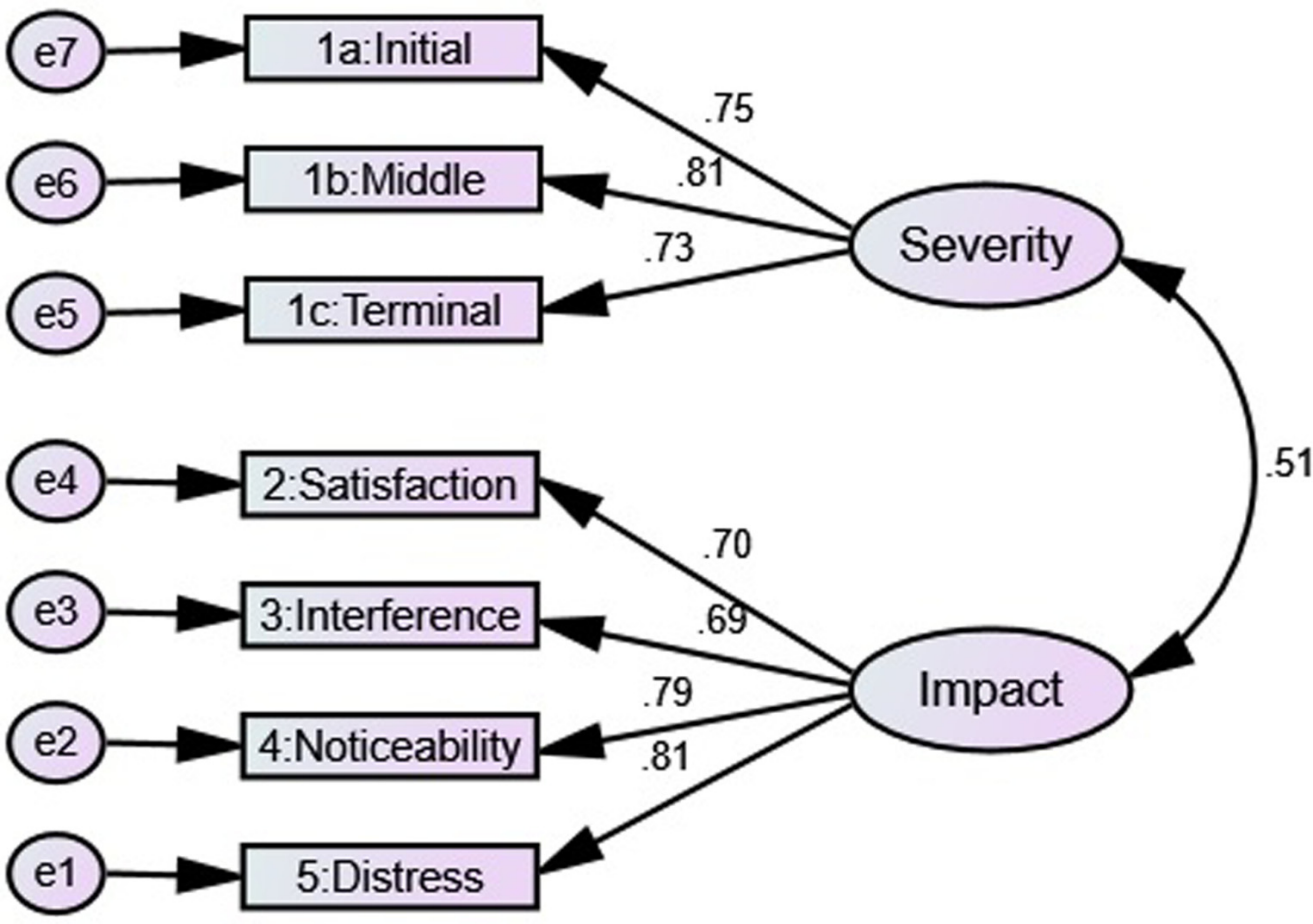
Confirmatory factor analysis for a two-factor model of Insomnia Severity Index (ISI) Arabic patients with chronic disease (*n*  =  504).

Confirmatory factor analyses: To determine the reliability if the factor structure from the PCA, a CFA was applied to the data from the replication sample (*n*  =  501). The PCA model (two-factor) was tested (χ^2^ (*df*  =  13)  =  17.94, *p*-value = .16, GFI  =  0.99, AGFI  =  0.98, CFI  =  0.99, TLI  =  0.99, RMSEA  =  0.03, and χ^2^/*df*  =  1.38), which indicated the absolute fit indices of the model (Figure 1).

**Figure 2.**
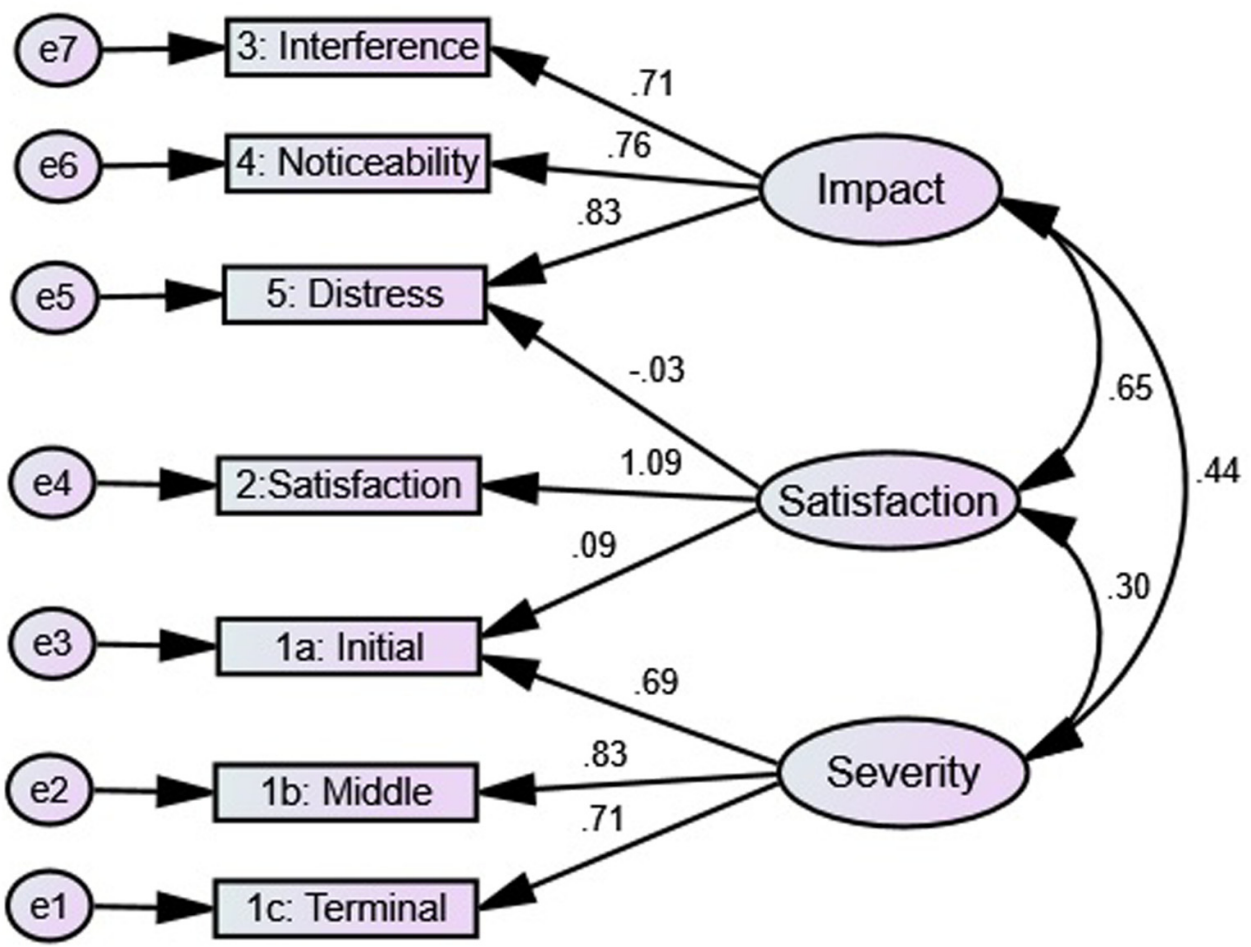
Confirmatory factor analysis for a three-factor model of Insomnia Severity Index (ISI) Arabic patients with chronic disease (*n*  =  504).

Additionally, the original English version of three-factor model suggested by Bastien et al., 2001 was tested. The three-factor model performed an absolute fit (χ^2^ [*df*  =  9] = 1.79, *p* = 0.06, GFI  =  0.99, AGFI  =  0.97, CFI  =  0.99, TLI  =  .99, RMSEA  =  0.04, and χ^2^/*df* = 1.79) (Figure 2).

## Discussion

Assessment of insomnia for Arabic chronic disease patients is necessary. However, a valid and reliable sleep instrument is needed to support clinical decision making with regard to interventions that can improve sleep. To the best of the present authors’ knowledge, this is the first study to examine the psychometric properties of the ISI in patients diagnosed with chronic diseases in Arabic populations. This study demonstrated that the Arabic version of the ISI tested in a chronic diseases’ population demonstrates adequate internal consistency as well as discriminant and construct validity. Confirmatory factor analysis supported the two-factor model. Although ISI items had floor effects, the total ISI score did not show either floor or ceiling effects. This indicates that the item analysis supported the content validity of the overall score of the scale (Lim et al., 2015).

There was an acceptable degree of internal consistency between the total score of the Arabic version of the ISI and the seven items, while Cronbach's alpha was 0.82. Previous studies have reported similar results in terms of clinical samples (Dieperink et al., 2020; Kraepelien et al., 2021). Manzar et al., (2021) conducted a systematic review of 20 studies that evaluated the measurement properties; they found Cronbach's alpha to be between 0.70 and 0.94, and the pooled overall Cronbach alpha reliability was 0.83. The original internal consistency proposed by (Bastien et al., 2001) was 0.74. The Arabic version of the ISI in non-clinical populations reported Alpha reliability of 0.84 (Suleiman & Yates, 2011). Thus, the findings related to internal consistency from this study were consistent with the published literature.

Additionally, the acceptable inter-item correlations provide further support for internal reliability consistency. All seven items of the ISI reported that the items total correlation coefficients were greater than 0.50. The items total correlation needed to be above 0.3, which was set as the minimum level of acceptance (Nunnally, 1994). The ISI appeared to be capable of discriminating between groups that differed in terms of gender, employment status, and comorbidities. Similar discriminant validity has been noted in psychometric studies among clinical samples (Gagnon et al., 2013; Morin et al., 2011).

The PCA of the ISI Arabic versions found two factor-structure loadings. The CFA of the current study found that a two-factor model which was labeled as “Factor I: Impact (2, 3, 4, and 5)” and “Factor II: Severity (1a, 1b, and 1c)” showed absolute fit indices. A similar model was found to have the best fit in studies involving a Portuguese clinical sample (Clemente et al., 2021), and an American sickle cell disease sample (Moscou-Jackson et al., 2016). In terms of clinical samples, previous studies conducted to evaluate the structure of the ISI show different model structures within the seven items. Two factors were found by Otte et al., 2019, Sadeghniiat-Haghighi et al., 2014, Savard et al., 2005, and a single factor by Kaufmann et al., 2019, Yusufov et al., 2021. This may be due to the differences in sample size and population characteristics. Another possible reason for the variation may be due to the differences in the cut-off values of the fit indices when it comes to determining the goodness of fit of the proposed model.

However, the results of the two-factor model of PCA in this study, indeed, are highly comparable with the result of CFA of the three factors’ models (Factor I “Severity”: items 1a, 1b, 1c; Factor II “Satisfaction”: item 2; Factor III “Impact”: items 3, 4, and 5) of the original English ISI (Bastien et al., 2001). The original three factors model had two cross-loading items (Figure 2). This may indicate a flaw in the factor structure (Asparouhov et al., 2015; Costello & Osborne, 2005). Notably, the satisfaction item uniquely clustered with impact items. Conceptually and empirically, the satisfaction item was a better fit for insomnia impact factors as it is measures the degree of happiness associated with sleep. Altogether, this study supports the two-factor structure of the Arabic ISI.

### Strengths and Limitation

There are several limitations to this study. The study employed a cross-sectional design. The stability of the instrument was not reported using test–retest reliability, therefore further assessment of test–retest reliability is needed. This study did not test concurrent validity. Consequently, further research should assess insomnia by ISI and another objective measure to identify the correlations between them. In addition, the study did not have a control group in the form of a non-clinical sample, the participants of which also complain of sleep disorders.

### Implication to Practice

The findings demonstrate that the Arabic version of the ISI demonstrates acceptable psychometric properties in chronic disease patients, and that it can be used to measure insomnia. The current results support the view that the ISI is best represented as a subjective measure for assessing insomnia in an Arabic population sample.

## Conclusion

The Arabic version of the ISI has acceptable psychometric properties in terms of internal consistency and discriminant validity. Therefore, the Arabic version of the ISI is a reliable and validated instrument to assess insomnia in Arabic chronic disease populations. Further research on the Arabic version should evaluate test–retest reliability using a longitudinal methodology.

## References

[bibr1-23779608221107278] Alshehri, M. M., Alenazi, A. M., Hoover, J. C., Alothman, S. A., Phadnis, M. A., Miles, J. M., Kluding, P. M., & Siengsukon, C. F. (2020). A comparison of diabetes self-care behavior in people with type 2 diabetes with and without insomnia symptoms. Acta Diabetologica, 57(6), 651–659. 10.1007/s00592-019-01470-y 31909434

[bibr2-23779608221107278] Angelhoff, C., Johansson, P., Svensson, E., & Sundell, A. L. (2020). Swedish translation and validation of the pediatric insomnia severity index. BMC Pediatrics, 20(1), 253. 10.1186/s12887-020-02150-5 32456677 PMC7249377

[bibr3-23779608221107278] Asparouhov T. Muthén B. Morin A. J. S. (2015). Bayesian structural equation modeling with cross-loadings and residual covariances: Comments on Stromeyer, et al. Journal of Management, 41(6), 1561–1577. 10.1177/0149206315591075

[bibr4-23779608221107278] Bartlett M. S. (1950). Tests of significance in factor analysis. British Journal of Statistical Psychology, 3(2), 77–85. 10.1111/j.2044-8317.1950.tb00285.x

[bibr5-23779608221107278] Bastien C. H. Vallières A. Morin C. M. (2001). Validation of the insomnia severity index as an outcome measure for insomnia research. Sleep Medicine, 2(4), 297–307. 10.1016/s1389-9457(00)00065-4 11438246

[bibr6-23779608221107278] Bean, H. R., Diggens, J., Ftanou, M., Weihs, K. L., Stanton, A. L., & Wiley, J. F. (2021). Insomnia and fatigue symptom trajectories in breast cancer: A longitudinal cohort study. Behavioral Sleep Medicine, 19(6), 814–827. 10.1080/15402002.2020.1869005 33470847

[bibr7-23779608221107278] Cho Y. W. Song M. L. Morin C. M. (2014). Validation of a Korean version of the insomnia severity index. Journal of Clinical Neurology (Seoul, Korea), 10(3), 210–215. 10.3988/jcn.2014.10.3.210 PMC4101097 25045373

[bibr8-23779608221107278] Clemente, V., Ruivo Marques, D., Miller-Mendes, M., Morin, C. M., Serra, J., & Allen Gomes, A. (2021). The European Portuguese version of the insomnia severity index. Journal of Sleep Research, 30(1), e13198. 10.1111/jsr.13198 32997368

[bibr9-23779608221107278] Costello A. Osborne J. (2005). Best practices in exploratory factor analysis: Four recommendations for getting the most from your analysis. Practical Assessment, Research, and Evaluation, 10(1), 1–9. 10.7275/jyj1-4868

[bibr10-23779608221107278] Da Costa, D., Allman, A.-A., Libman, E., Desormeau, P., Lowensteyn, I., & Grover, S. (2017). Prevalence and determinants of insomnia after a myocardial infarction. Psychosomatics, 58(2), 132–140. 10.1016/j.psym.2016.11.002 28104338

[bibr11-23779608221107278] Dieperink, K. B., Elnegaard, C. M., Winther, B., Lohman, A., Zerlang, I., Möller, S., & Zangger, G. (2020). Preliminary validation of the insomnia severity index in Danish outpatients with a medical condition. Journal of Patient-Reported Outcomes, 4(18), 1–10. 10.1186/s41687-020-0182-6 PMC7052094 32124114

[bibr12-23779608221107278] Frøjd, L. A., Munkhaugen, J., Moum, T., Sverre, E., Nordhus, I. H., Papageorgiou, C., & Dammen, T. (2021). Insomnia in patients with coronary heart disease: Prevalence and correlates. Journal of Clinical Sleep Medicine, 17(5), 931–938. 10.5664/jcsm.9082 PMC8320477 33399066

[bibr13-23779608221107278] Gagnon, C., Bélanger, L., Ivers, H., & Morin, C. M. (2013). Validation of the insomnia severity index in primary care. Journal of the American Board of Family Medicine: JABFM, 26(6), 701–710. 10.3122/jabfm.2013.06.130064 24204066

[bibr14-23779608221107278] Hair, J. F., Black, W. C., Babin, B. J., & Anderson, R. E. (2014). Multivariate data analysis: Pearson new international edition (7th ed). Pearson Higher Ed.

[bibr15-23779608221107278] Horn J. L. (1965). A rationale and test for the number of factors in factor analysis. Psychometrika, 30(2), 179–185. 10.1007/BF02289447 14306381

[bibr16-23779608221107278] Kaiser H. F. (1970). A second generation little Jiffy. Psychometrika, 35(4), 401–415. 10.1007/BF02291817

[bibr17-23779608221107278] Kaufmann, C. N., Orff, H. J., Moore, R. C., Delano-Wood, L., Depp, C. A., & Schiehser, D. M. (2019). Psychometric characteristics of the insomnia severity index in veterans with history of traumatic brain injury. Behavioral Sleep Medicine, 17(1), 12–18. 10.1080/15402002.2016.1266490 28098495 PMC5740012

[bibr18-23779608221107278] Kayaba, M., Sasai-Sakuma, T., Takaesu, Y., & Inoue, Y. (2021). The relationship between insomnia symptoms and work productivity among blue-collar and white-collar Japanese workers engaged in construction/civil engineering work: A cross-sectional study. BMC Public Health, 21(1), 1244. 10.1186/s12889-021-11273-y 34182963 PMC8240247

[bibr19-23779608221107278] Kline R. B. (2015). Principles and practice of structural equation modeling (Fourth). The Guilford Press.

[bibr20-23779608221107278] Koopman, A. D. M., Beulens, J. W., Dijkstra, T., Pouwer, F., Bremmer, M. A., van Straten, A., & Rutters, F. (2020). Prevalence of insomnia (symptoms) in T2D and association with metabolic parameters and glycemic control: Meta-analysis. The Journal of Clinical Endocrinology & Metabolism, 105(3), 614–643. 10.1210/clinem/dgz065 PMC7110921 31603475

[bibr21-23779608221107278] Kraepelien M. Blom K. Forsell E. , et al. (2021). A very brief self-report scale for measuring insomnia severity using two items from the insomnia severity Index - development and validation in a clinical population. Sleep Medicine, 81, 365–374. 10.1016/j.sleep.2021.03.003 33813233

[bibr22-23779608221107278] Lahan V. Gupta R. (2011). Translation and validation of the insomnia severity index in Hindi language. Indian Journal of Psychological Medicine, 33(2), 172–176. 10.4103/0253-7176.92060 22345845 PMC3271495

[bibr23-23779608221107278] Li X. Sotres-Alvarez D. Gallo L. C. , et al. (2021). Associations of sleep-disordered breathing and insomnia with incident hypertension and diabetes. The Hispanic community health study/study of Latinos. American Journal of Respiratory and Critical Care Medicine, 203(3), 356–365. 10.1164/rccm.201912-2330OC PMC7874314 32758008

[bibr24-23779608221107278] Lim C. R. Harris K. Dawson J. , et al. (2015). Floor and ceiling effects in the OHS: An analysis of the NHS PROMs data set. BMJ Open, 5(7), e007765. 10.1136/bmjopen-2015-007765 PMC4521553 26216152

[bibr25-23779608221107278] Lovato N. Lack L. (2019). Insomnia and mortality: A meta-analysis. Sleep Medicine Reviews, 43, 71–83. 10.1016/j.smrv.2018.10.004 30529432

[bibr26-23779608221107278] Manzar M. D. Jahrami H. A. Bahammam A. S. (2021). Structural validity of the insomnia severity index: A systematic review and meta-analysis. Sleep Medicine Reviews, 60, 101531. 10.1016/j.smrv.2021.101531 34428679

[bibr27-23779608221107278] Morin, C. M., Belleville, G., Bélanger, L., & Ivers, H. (2011). The insomnia severity index: Psychometric indicators to detect insomnia cases and evaluate treatment response. Sleep, 34(5), 601–608. 10.1093/sleep/34.5.601 21532953 PMC3079939

[bibr28-23779608221107278] Morlock A. Dobrescu R. (2018). Self-reported insomnia symptoms and health related quality of life in the US adult population. Value in Health, 21, S208. 10.1016/j.jval.2018.04.1427

[bibr29-23779608221107278] Moscou-Jackson, G., Allen, J., Smith, M. T., & Haywood, C. (2016). Psychometric validation of the insomnia severity index in adults with sickle cell disease. Journal of Health Care for the Poor and Underserved, 27(1), 209–218. 10.1353/hpu.2016.0010 27217712 PMC4874249

[bibr30-23779608221107278] Nunnally J. C. (1994). Psychometric theory (3rd ed). McGraw-Hill.

[bibr31-23779608221107278] Olaithe M. Ree M. McArdle N. , et al. (2021). Cognitive dysfunction in insomnia phenotypes: Further evidence for different disorders. Frontiers in Psychiatry, 12(688672), 1–11. 10.3389/fpsyt.2021.688672 PMC8326515 34349682

[bibr32-23779608221107278] Otte, J. L., Bakoyannis, G., Rand, K. L., Ensrud, K. E., Guthrie, K. A., Joffe, H., McCurry, S. M., Newton, K. M., & Carpenter, J. S. (2019). Confirmatory factor analysis of the insomnia severity index (ISI) and invariance across race: A pooled analysis of MsFLASH data. Menopause, 26(8), 850–855. 10.1097/GME.0000000000001343 PMC6663566 30994570

[bibr33-23779608221107278] Robertson, M. E., McSherry, F., Herndon, J. E., & Peters, K. B. (2016). Insomnia and its associations in patients with recurrent glial neoplasms. SpringerPlus, 5(1), 823. 10.1186/s40064-016-2578-6 27390663 PMC4916119

[bibr34-23779608221107278] Sadeghniiat-Haghighi, K., Montazeri, A., Khajeh-Mehrizi, A., Nedjat, S., & Aminian, O. (2014). The insomnia severity index: Cross-cultural adaptation and psychometric evaluation of a Persian version. Quality of Life Research: An International Journal of Quality of Life Aspects of Treatment, Care and Rehabilitation, 23(2), 533–537. 10.1007/s11136-013-0489-3 23912857

[bibr35-23779608221107278] Savard, M.-H., Savard, J., Simard, S., & Ivers, H. (2005). Empirical validation of the insomnia severity index in cancer patients. Psycho-Oncology, 14(6), 429–441. 10.1002/pon.860 15376284

[bibr36-23779608221107278] Schulte T. Hofmeister D. Mehnert-Theuerkauf A. , et al. (2021). Assessment of sleep problems with the insomnia severity index (ISI) and the sleep item of the patient health questionnaire (PHQ-9) in cancer patients. Supportive Care in Cancer. 29(12), 7377–7384. 10.1007/s00520-021-06282-x PMC8550304 34050799

[bibr37-23779608221107278] Sellami M. Hamdi O. Miladi S. , et al. (2021). Pos0159-Hpr impact of sleep disturbances on elderly patients with rheumatoid arthritis. Annals of the Rheumatic Diseases, 80(Suppl 1), 292–292. 10.1136/annrheumdis-2021-eular.408

[bibr38-23779608221107278] Shapour B. Gang C. (2013). Reliability and validity of the Chinese translation of insomnia severity index and comparison with Pittsburgh sleep quality index. Malaysian Journal of Psychiatry, 22(2). 3–9.

[bibr39-23779608221107278] Siebmanns, S., Johansson, P., Ulander, M., Johansson, L., Andersson, G., & Broström, A. (2021). The effect of nurse-led internet-based cognitive behavioural therapy for insomnia on patients with cardiovascular disease: A randomized controlled trial with 6-month follow-up. Nursing Open, 8(4), 1755–1768. 10.1002/nop2.817 33609425 PMC8186676

[bibr40-23779608221107278] Smith T. J. Wilson M. Karl J. P. , et al. (2018). Impact of sleep restriction on local immune response and skin barrier restoration with and without “multinutrient” nutrition intervention. Journal of Applied Physiology, 124(1), 190–200. 10.1152/japplphysiol.00547.2017 28912361

[bibr41-23779608221107278] Suleiman K. H. Yates B. C. (2011). Translating the insomnia severity index into Arabic. Journal of Nursing Scholarship, 43(1), 49–53. https://doi.org/10.1111/j.1547-5069.2010.01374.x 21342424 10.1111/j.1547-5069.2010.01374.x

[bibr42-23779608221107278] Yusufov M. Recklitis C. Zhou E. S. , et al. (2021). A population-based psychometric analysis of the insomnia severity index in black women with and without a history of cancer. Journal of Sleep Research, 31(e13421), 1–11. 10.1111/jsr.13421 PMC13150567 34128264

